# Recent Advances in the Health Benefits and Application of Tangerine Peel (*Citri Reticulatae Pericarpium*): A Review

**DOI:** 10.3390/foods13131978

**Published:** 2024-06-23

**Authors:** Minke Shi, Qihan Guo, Zhewen Xiao, Sarengaowa, Ying Xiao, Ke Feng

**Affiliations:** 1 Medical Sciences Division, Macau University of Science and Technology, Macao 999078, China; minkeshi8@gmail.com (M.S.); a1025732821@gmail.com (Q.G.); 13681245239@163.com (Z.X.); yxiao@must.edu.mo (Y.X.); 2 School of Life Science, Zhuhai College of Science and Technology, Zhuhai 519041, China; kuailexiaosa@sina.com

**Keywords:** tangerine peel, *Citri Reticulatae Pericarpium*, chemical composition, health benefits, food applications

## Abstract

Citrus fruits, renowned for their abundant of phytochemicals and bioactive compounds, hold a prominent position as commercially grown fruits with health-promoting properties. In this context, tangerine peel (*Citri Reticulatae Pericarpium*, CRP) is garnering attention as a byproduct of citrus fruits. Within the framework of the circular economy, CRP has emerged as a focal point due to its potential health benefits. CRP, extracted from *Citrus reticulata* cv. and aged for over three years, has attracted increasing attention for its diverse health-promoting effects, including its anticancer, cardiovascular-protecting, gastrointestinal-modulating, antioxidant, anti-inflammatory, and neuroprotective properties. Moreover, CRP positively impacts skeletal health and various physiological functions. This review delves into the therapeutic effects and molecular mechanisms of CRP. The substantial therapeutic potential of CRP highlights the need for further research into its applications in both food and medicine. As a value-added functional ingredient, CRP and its constituents are extensively utilized in the development of food and health supplements, such as teas, porridges, and traditional medicinal formulations.

## 1. Introduction

In China, citrus fruits are widely cultivated, and they contain valuable phytochemicals. Moreover, their areas of cultivation and the extent of their application are under considerable scrutiny. As a member of the Rutaceae family, citrus is regarded as one of the largest plant species, with a wide distribution across tropical, subtropical, and temperate regions globally due to natural or artificial hybridization, resulting in numerous different varieties and hybrids [1]. In Asian countries, particularly in China, Japan, and South Korea, citrus peel holds significant importance in traditional medicine [2]. In China, common citrus varieties are used to produce dried tangerine peel, with its extract widely employed in thousands of traditional medicine prescriptions. It is believed to confer health benefits against various diseases. *Citri Reticulatae Pericarpium* (CRP), commonly known as dried tangerine peel in English, is a widely used traditional Chinese medicine (TCM) derived from the mature peels of citrus plants belonging to the Rutaceae family, particularly various cultivated varieties of *Citrus Reticulata* [3]. Its use was first documented in “Shennong Ben Cao Jing”, an ancient Chinese medicinal text, and has a history spanning thousands of years in China [4]. CRP is extensively utilized in TCM formulations and is also added as a flavoring agent in foods throughout China due to its distinct pharmacological properties, low toxicity, and high efficacy [5]. As a significant medicinal herb, CRP is renowned for its various therapeutic effects, including tonifying the spleen, promoting qi circulation, dispelling dampness, and resolving phlegm [6]. Qi circulation in TCM refers to the movement and distribution of refined nutritious substances and energy throughout the body. It is essential for maintaining life activities and the functions of the Zang-Fu organs. Qi circulation ensures the proper functioning of the body’s systems, promoting health and balance [7]. It is widely distributed in regions such as Guangdong, Fujian, Sichuan, Zhejiang, Jiangxi, and Hunan provinces in China [6]. Varieties of CRP, such as Guangchenpi (*Citrus reticulata* “Chachi”), Chuanchenpi (*C. reticulata* “Dahongpao”), Zhechenpi (*C. reticulata* “Unshiu”), and Jianchenpi (*C. reticulata* “Tangerina”), are documented in the Chinese Pharmacopoeia [8]. CRP, particularly the dried mature peels of a variety known as “Chacha” from Xinhui District, Guangdong Province, known as Guangchenpi (GCP) in Chinese, is highly esteemed for its unique efficacy and is considered a prestigious medicinal product of the region [9].

**Figure 1:**
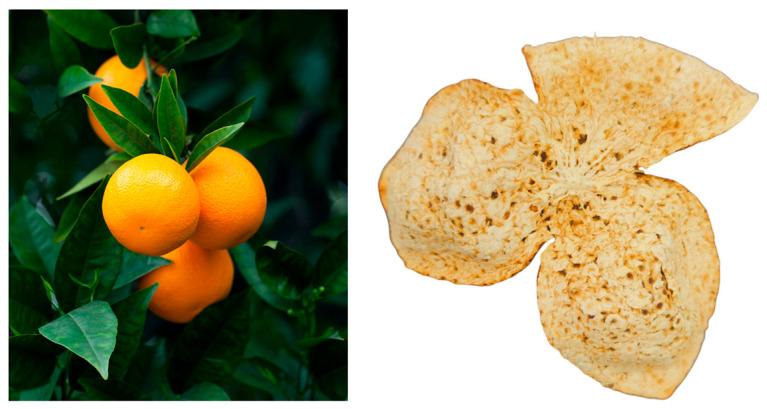
*Citrus Reticulata*.

Distinguishing it from regular oranges, this fruit exhibits a slightly concave shape with distinct columnar markings (Figure 1). Sometimes, it features a small navel or a radiating furrow around the stem end. Additionally, it measures approximately 4.6–5.9 cm in horizontal diameter and 6.3–7.1 cm in vertical diameter, weighs around 100–138 g, has a thickness of 2.7–3.3 mm, contains 15–25 seeds, and has multiple embryos [6]. The cultivation of *Citrus reticulata* for CRP extraction focuses solely on obtaining its peels for medicinal purposes rather than for consumption, as the fruit itself tastes bitter, and its peels are thick and hard [10]. Mature fruits are typically harvested in late autumn and early winter, after which their peels are removed and dried under ventilated or low-temperature conditions. To process CRP into medicinal products, impurities must be removed, and procedures such as washing, cutting, and drying are employed. The product is presented in irregular strips or fine shreds [11].

CRP is extensively used in traditional medicine for the treatment of conditions like vomiting, nausea, and anemia [12]. Its applications have expanded into broader domains, such as the food and cosmetics industries, where it serves as a principal component in dietary supplements, tea, and even essential oils in cosmetics, showcasing its multifaceted potential applications and benefits across different sectors [12]. Despite the possibility of economic and environmental issues arising from the fermentation and microbial spoilage of citrus peel, widespread applications in medicine, food, and cosmetics continue to highlight its value as a valuable byproduct of the citrus industry [13]. Its accessibility, cost-effectiveness, and potential as a substitute for functional ingredients and chemical preservatives in food, the peel of citrus species has rich dietary fiber, minerals, and antioxidant properties [14]. This review aims to provide a comprehensive overview of health benefits and applications of CRP in food. We will explore the traditional health benefits associated with CRP. This review intends to lay the foundation for further research and development, facilitating the sustainable utilization of CRP across various sectors.

## 2. Health Benefits

**Figure 2:**
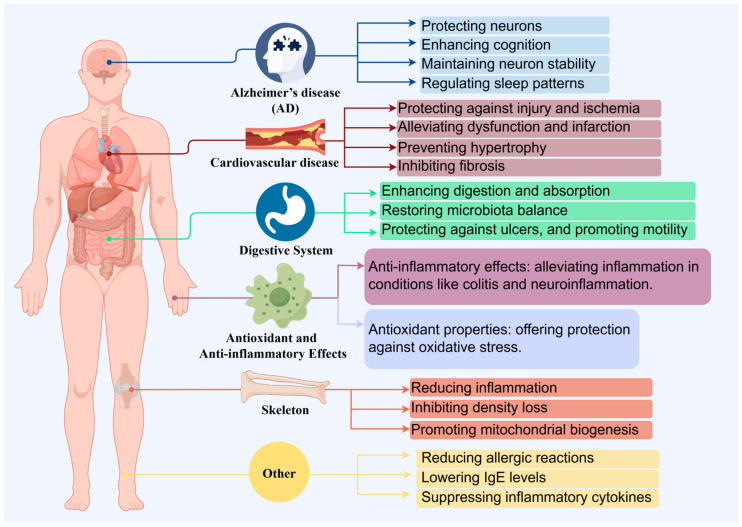
The health benefits of *Citri Reticulatae Pericarpium*.

Citrus fruits are widely appreciated for their distinctively sweet and tangy flavor profile. From this, tangerine peel is also a major byproduct of the citrus processing industry [15], and recently, tangerine peel has garnered attention due to its health benefits. Through a prolonged natural aging and storage process, tangerine peel transforms into mature dried peel known as “Chenpi” (CRP) [16]. CRP is a renowned medicinal food that is extensively produced in provinces such as Guangdong, Fujian, Sichuan, Chongqing, and Zhejiang in China [2]. Chemical analysis of CRP has identified approximately 140 compounds, including flavonoids, volatile oils, and alkaloids [17]. These citrus extracts are reported to possess various bioactivities, including anticancer [18], anti-inflammatory [12,19], and antioxidant properties (Figure 2) [20]. Thus, starting from tangerine peel, the prolonged aging and storage process leads to the formation of CRP, a product rich in flavonoids and other bioactive compounds. CRP is extensively used in the food and pharmaceutical industries, not only due to its unique flavor but also due to its potential health benefits, including anticancer, cardiovascular diseases effect, digestive system effect, anti-inflammatory, antioxidant and skeleton effect (Table 1) [21,22].

### 2.1. Anticancer Effects

**Figure 3:**
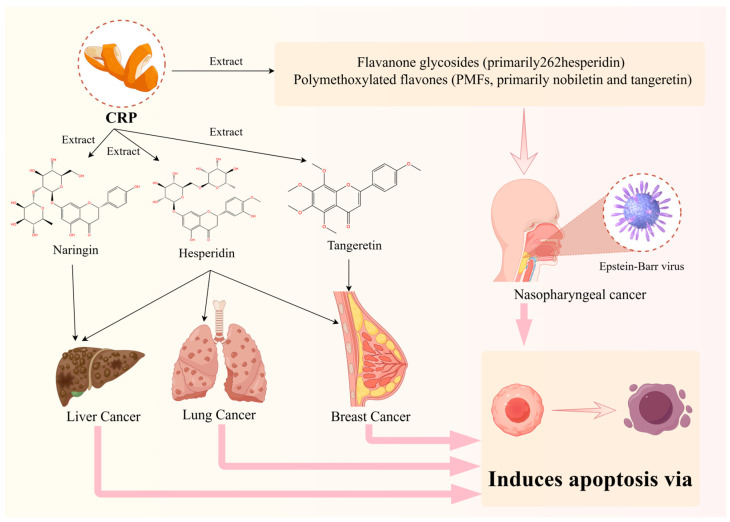
Efficacy of representative *Citri Reticulatae Pericarpium* extracts for cancer.

Cancer originates from the clonal expansion and development of aberrant cells within the body [57]. With the changing prevalence and distribution of major risk factors, the global incidence and mortality rates of cancer are rapidly increasing [4]. Conventional cancer treatment methods include surgery, radiation therapy, chemotherapy, targeted therapy, hormone therapy, and immunotherapy [58]. However, current treatments often come with a variety of physical and psychological side effects, significantly affecting prognosis and life expectancy. As a botanical medicine, CRP has various pharmacological effects and can be used alone or in combination with other traditional Chinese medicines to form many well-known classic prescriptions. It is widely used in the clinical treatment of various systemic diseases, especially for its contribution to lung cancer, nasopharyngeal cancer, liver cancer, and breast cancer treatment (Figure 3). In recent years, significant attention has been given to clinical and laboratory research on traditional Chinese medicine (TCM) for cancer treatment [59]. The distinct efficacy and safety profile of Chinese medicine make it a unique and noteworthy approach in the field of cancer therapy. CRP, as a substance with medicinal and dietary origins, plays two roles: it is a traditional Chinese medicine and a food source. This dual purpose underscores the potential of CRP in the field of cancer treatment.

#### 2.1.1. Lung Cancer

Ranked as the second-most-prevalent cancer globally, lung cancer claimed the top position for cancer-related mortality in 2020, with incidence and death rates approximately double for men compared to women [60]. CRP is good at treating respiratory system diseases, and pharmacological researchers have carried out an in-depth exploration of the treatment of lung cancer [61]. Hesperidin, a flavonoid found in CRP, has been demonstrated in A549 human NSCLC cell models to downregulate the expression of matrix metalloproteinases (MMPs), enhance antioxidant status, and counteract nicotine toxicity, thereby mitigating smoking-induced lung cancer suppression [23]. The antioxidant properties of hesperidin also exhibited inhibitory effects on tumor cell proliferation in a benzo(a)pyrene-induced lung cancer mouse model [62]. Another study suggested that hesperidin alleviates non-small cell lung cancer (NSCLC) by suppressing cell proliferation and inducing apoptosis through the miR-132/ZEB2 signaling pathway [24]. Hesperidin induces apoptosis via the mitochondrial pathway; upregulates the expression of P21 and P53, triggering G0/G1 phase arrest in A549 cells; and downregulates cyclin D1, thereby inhibiting cell proliferation [4]. Furthermore, it has been shown that naringin, another important natural compound of CRP, attenuated EGF-induced MUC5AC mucin and mRNA overexpression by inhibiting the synergistic activity of the MAPKs/AP-1 and IKKs/IκB/NF-κB signaling pathways when using A549 cells as a study subject [27]. Naringin exhibits the ability to inhibit the PI3K/AKT/mTOR and NF-κB pathways and activate miR-126 expression in H69 cells, thereby preventing cell growth and inducing apoptosis in SCLC cells [28]. In summary, CRP holds significant promise in the realm of lung cancer treatment, especially considering its capacity to address respiratory system ailments.

#### 2.1.2. Nasopharyngeal Cancer

Nasopharyngeal carcinoma (NPC) is a distinctive malignant tumor originating from the nasopharyngeal epithelium. NPC is closely related to Epstein–Barr virus, which is one of the major causes of NPC [63]. Natural products derived from traditional Chinese medicine exhibit potential as antitumor drugs due to their low toxicity and minimal side effects [30]. The major components of CRP are flavonoids, which are generally categorized into two groups: flavanone glycosides (primarily hesperidin) and polymethoxylated flavones (PMFs, primarily nobiletin and tangeretin). PMFs consist of nobiletin, tangeretin, 3,5,6,7,8,3′,4′-heptamethoxyflavone, and 5-hydroxy-6,7,8,3′,4′-pentamethoxyflavone [64]. Over the past decade, extensive research has unveiled multiple therapeutic effects of nobiletin, particularly its noteworthy antitumor capabilities, particularly in nasopharyngeal cancer. PARP-2, or Poly (ADP-ribose) polymerase-2, is an enzyme crucial for repairing DNA damage, particularly in response to breaks in DNA strands. SIRT1, known as Sirtuin 1, functions as a histone deacetylase enzyme, relying on NAD+ for its activity [65]. It serves as a downstream target of PARP and is involved in various cellular processes, including metabolism and DNA repair [66]. Additionally, AMPK, or AMP-activated protein kinase, acts as a central regulator of energy metabolism. It is activated through phosphorylation and is one of the substrates of SIRT1 [67]. AMPK plays a pivotal role in maintaining cellular energy homeostasis by promoting ATP production and inhibiting energy-consuming processes when cellular energy levels are low [68]. CRP has been found to regulate the PARP-2/SIRT1/AMPK signaling pathway, leading to growth inhibition and apoptosis induction in C666-1 cells (C666-1 is the exclusive one that still retains the natural EBV) [30]. Specifically, CRP inhibits the expression of PARP-2, and the partial alleviation of its impact on C666-1 cells by PARP-2 overexpression suggests the potential role of PARP-2 in the growth inhibition and apoptosis induced by CRP [35]. Furthermore, CRP treatment activates the SIRT1 and AMPK signaling pathways, involving AMPK phosphorylation and the inhibition of the mTOR signaling pathway [69]. This indicates that CRP may induce cell apoptosis by activating AMPK, providing crucial clues to unraveling its anticancer mechanisms. Notably, nobiletin-induced apoptosis in SNU-16 cells is regulated by intracellular endoplasmic reticulum stress-mediated protective autophagy [70]. Studies have reported the inhibitory effects of nobiletin on the invasion and migration of HONE-1 and NPC-BM, human nasopharyngeal carcinoma (NPC) cell lines [30]. Among NPC-derived cell lines, C666-1, retaining natural Epstein–Barr virus (EBV), stands out as a crucial and representative tool for evaluating antitumor activity against NPC [30]. CRP and its primary component nobiletin exhibit significant potential in nasopharyngeal carcinoma research. This discovery provides a scientific basis for the application of CRP and its active components in nasopharyngeal carcinoma treatment, offering promising prospects for future in-depth research and clinical applications.

#### 2.1.3. Liver Cancer

The investigation into the therapeutic potential of CRP for liver cancer primarily concentrates on hepatocellular carcinoma (HCC). Hesperidin, as a component found in the CRP, plays a crucial role in combating the invasiveness of HCC cells. Its mechanism primarily involves inhibiting NF-κB and AP-1 activities, consequently leading to the downregulation of MMP-9 expression and secretion in acetaldehyde- and 12-O-Tetradecanoylphorbol-13-acetate (TPA)-induced HCC [71]. The induction of apoptosis in HepG2 cells through the upregulation of the pro-apoptotic protein BAX represents an effective mechanism employed by hesperidin and naringin to hinder the progression of liver cancer [33].

#### 2.1.4. Breast Cancer

Breast cancer poses a significant threat to women worldwide, with the highest diagnosis rate in this demographic, impacting both survival and quality of life. In recent studies, nobiletin was identified as a promising agent in inhibiting the ERK1/2 and PI3K/AKT pathways, concurrently suppressing the growth of TNBC MDA-MB-468 cells. Its antitumor effects are attributed to the combination of anti-proliferative actions and the induction of apoptosis [72]. Tangeretin, another compound under investigation, inhibits breast cancer cell metastasis by targeting key factors such as TP53, PTGS2, MMP9, and PIK3CA, while also modulating the PI3K/AKT pathway [35]. Furthermore, tangeretin exhibits the potential to inhibit the formation of breast cancer stem cells (BCSCs) by acting on the Stat3/Sox2 signaling pathway, offering a potential therapeutic strategy for breast cancer and BCSCs [36]. Hesperidin, through concentration-dependent cytotoxicity, manifests inhibitory effects on the human breast cancer cell line MCF-7, inducing apoptosis and causing DNA damage [4]. Naringin, on the other hand, hampers cell proliferation, promotes apoptosis, and induces G1 cycle arrest by regulating the β-catenin pathway. This regulatory action suppresses the growth potential of TNBC cells [72]. Moreover, hesperidin demonstrates inhibitory activity against the proliferation of MCF-7-GFP-Tubulin cells, combats drug-resistant cancer cells, and mitigates cell migration in MDA-MB 231 cells [4]. These findings collectively underscore the multifaceted potential of flavonoid compounds from CRP in terms of addressing various aspects of breast cancer. These promising results provide broad prospects for further research and therapeutic development in the field.

### 2.2. Cardiovascular Disease Effects

Cardiovascular diseases (CVDs) encompass a group of disorders affecting the heart and its intricate vascular system. Examples of CVDs include coronary artery disease, stroke, peripheral vascular diseases, heart failure, and arterial aneurysms, with most of these conditions closely associated with the development and progression of atherosclerosis [73]. Numerous epidemiological studies have demonstrated a positive correlation between the intake of citrus flavonoids and reduced cardiovascular mortality [74]. Both in vitro and in vivo studies have also revealed that CRP and its principal active constituents can effectively treat CVD [75]. CRP, derived from *Citrus reticulata* and its cultivar, is characterized by a dry and mature pericarp [76]. Rich in beneficial components like flavonoids, alkaloids, and volatile oils, CRP has been extensively studied for its health-promoting properties [6]. Noteworthy compounds identified in CRP include hesperidin, nobiletin, naringin, and naringenin, all recognized for their cardioprotective effects [76]. Hesperidin, hesperetin, and their derivatives exhibit positive impacts against myocardial injury, cardiac remodeling, as well as myocardial ischemia and infarction [77]. Studies have indicated that hesperidin plays a role in inhibiting cardiomyocyte apoptosis and reducing oxidative stress damage by upregulating Proliferator-Activated Receptor Gamma (PPARγ) expression [42]. Nobiletin has demonstrated effectiveness in alleviating myocardial dysfunction in diabetic rat models and attenuating myocardial ischemia and reperfusion injury [78]. It also inhibits endoplasmic reticulum stress-associated apoptosis through the regulation of the PI3K/AKT signaling pathway [78]. Naringenin, a flavonoid found in citrus fruits like grapefruits, is recognized for its beneficial effects in reducing ischemic damage. It achieves this by activating mitochondrial BK (Big Potassium) channels [42]. These channels, located in the inner mitochondrial membrane, play a crucial role in maintaining cellular energy metabolism and protecting against ischemic injury. Naringenin’s activation of these channels helps regulate calcium levels, maintain mitochondrial membrane potential, inhibit apoptosis, and reduce oxidative stress, thereby preserving mitochondrial function and cell integrity during ischemic conditions [43]. Additionally, naringin has been found to protect against lipopolysaccharide (LPS)-induced myocardial dysfunction in rats by regulating the PI3K/AKT/NF-κB pathway [44]. The collective findings underscore the potential cardiovascular benefits of CRP and its individual components. CRP can prevent pathological cardiac hypertrophy induced by angiotensin II (Ang II) through the upregulation of PPARγ, as demonstrated in studies by Ni et al. [45]. The therapeutic application of tangerine peel (CRP) has been shown to improve cardiac function and significantly alleviate myocardial hypertrophy and cardiac fibrosis [45]. A key molecular mechanism involves the activation of PPARγ by tangerine peel, with Peroxisome Proliferator-Activated Receptors (PPARs) playing a crucial role in regulating cardiac metabolism and pathological cardiac hypertrophy [79]. Results from CRP treatment have indicated an upregulation of PPARγ expression levels, thereby inhibiting the progression of pathological cardiac hypertrophy [45]. Studies at the cellular and molecular levels have further revealed the anti-fibrotic effects of tangerine peel. By downregulating the expression of fibrosis-related genes, CRP effectively retards the development of cardiac fibrosis [45]. In summary, tangerine peel demonstrates significant potential in preventing and mitigating pathological cardiac hypertrophy by modulating the activation of PPARγ and inhibiting fibrosis. This provides valuable insights for the future development of novel approaches for cardiovascular disease therapy.

### 2.3. Effect on the Digestive System

In the theory of traditional Chinese medicine, spleen deficiency is a common clinical syndrome characterized by symptoms such as emaciation, loss of appetite, epigastric pain, bloating, fatigue, sallow complexion, and loose stools [80]. Modern research has indicated that spleen deficiency is a comprehensive manifestation of various functions, including food digestion, nutrient absorption, energy metabolism, and immune system decline [81]. CRP is a crucial traditional Chinese medicine utilized in the treatment of conditions associated with spleen deficiency [80]. The aim refers to the study described in the paragraph, which explores the mechanisms by which CRP alleviates spleen deficiency-related diseases through integrating pharmacology and gut microbiota analysis. The study evaluated CRP’s therapeutic effects on spleen deficiency symptoms in reserpine-treated rats, investigated its impact on gut microbiota composition, identified bioactive compounds using ultra-high performance liquid chromatography-quadrupole-time of flight tandem mass spectrometry, and utilized network pharmacology to elucidate its therapeutic pathways [82]. And then, CRP demonstrated effectiveness in relieving key symptoms of spleen deficiency, including compromised digestion and absorption capabilities, as well as disruptions in gastrointestinal hormones, immune cytokines, and oxidative stress [80]. Subsequently, high-throughput 16S rRNA gene sequencing revealed that CRP, by modulating the gut microbiota, not only upregulated the production of short-chain fatty acids and anti-inflammatory bacteria but also downregulated certain bacteria exacerbating spleen deficiency [80]. This finding ultimately facilitated the restoration of gut microbiota balance in rats with spleen deficiency, thereby contributing to improved digestive function of the spleen. Additionally, the combined treatment of rats with hesperidin and lycopene from CRP demonstrated a significant improvement in ulcer conditions, including gastric pH, volume of gastric content, total acidity, and ulcer index [46]. Furthermore, hesperidin exhibited a protective effect against all gastric injuries induced by ethanol administration in rats, partially attributed to its antioxidant properties [83]. Simultaneously, CRP’s regulatory effect on the digestive system is primarily manifested in its modulation of the gastrointestinal tract. Experimental evidence confirms that CRP has a dual action in promoting gastrointestinal motility and inhibiting intestinal smooth muscle contraction [6]. This bidirectional influence may alter gastrointestinal function by impacting the secretion of digestive organs or directly affecting intestinal smooth muscle [6]. Hesperidin promotes gastrointestinal motility by increasing levels of acetylcholine (ACh) and motilin (MTL), while decreasing levels of Substance P (SP) and vasoactive intestinal peptide (VIP). Additionally, the ethyl acetate extract of CRP also demonstrates the ability to enhance gastrointestinal motility [84].

### 2.4. Antioxidant and Anti-Inflammatory Effects

CRP demonstrates a potent anti-inflammatory impact attributed to hesperidin, nobiletin, naringenin, and tangeretin, as reported by Chen et al. [85]. This anti-inflammatory effect operates by reducing the secretion of proinflammatory cytokines such as TNF-α, IL-1β, and IL-6. Moreover, CRP inhibits the expression of inducible nitric oxide synthase (iNOS) and cyclooxygenase (COX-2) genes, leading to a decrease in production in RAW 264.7 macrophages and LPS-induced microglia. Significantly, a study conducted by Lee et al. [50] revealed that CRP inhibits the activities, mRNA levels, and protein levels of MMP-3 and MMP-8 while enhancing the expression of tissue inhibitor of MMP-2. This indicates CRP’s potential to ameliorate experimental colitis and neuroinflammation, as highlighted by Ho and Kuo [47] and Xiong et al. [86]. However, it is emphasized that further in vivo pharmacological studies are necessary to validate the anti-inflammatory efficacy of CRP. In addition to its anti-inflammatory properties, CRP’s flavonoids, including hesperidin, nobiletin, and tangeretin, exhibit diverse antioxidant capabilities. Su et al. [87] reported that these flavonoids are proficient in scavenging 1,1-diphenyl-2-picrylhydrazyl (DPPH), hydroxyl radicals, superoxide anion radicals, and hydrogen peroxide, as well as chelating ferrous ions. This dual functionality highlights the potential of CRP as a therapeutic agent with both anti-inflammatory and antioxidant properties. Furthermore, these flavonoids have been shown to inhibit lipid peroxidation and protect cells from oxidative stress-induced damage. Hesperidin, for example, has been demonstrated to enhance the activities of antioxidant enzymes such as superoxide dismutase (SOD) and catalase [47]. Nobiletin and tangeretin also exhibit neuroprotective effects by reducing oxidative stress in neuronal cells. The antioxidant mechanisms of CRP flavonoids involve direct scavenging of free radicals, modulation of antioxidant enzyme activities, and metal chelation, which collectively contribute to their protective effects against oxidative damage. This multifaceted antioxidant activity positions CRP as a promising therapeutic candidate for conditions associated with oxidative stress and inflammation [87].

### 2.5. Alzheimer’s Disease (AD)

Alzheimer’s disease (AD), characterized by the presence of amyloid-β (Aβ) plaques and neurofibrillary tangles, along with neurodegeneration, is the most common form of age-related neurodegenerative disease [88]. Given the current limitations of available treatments that can significantly alter the progression of these diseases, there is a compelling need for effective therapeutic and preventive interventions. During our exploration of natural substances exhibiting anti-dementia and neuroprotective properties, we identified nobiletin, a polymethoxylated flavone derived from CRP [89]. Nobiletin demonstrated the capacity to ameliorate cognitive deficits and address pathological features associated with AD, including Aβ pathology, hyperphosphorylation of tau, and oxidative stress, as observed in animal models of AD [47]. These findings suggest that nobiletin holds promise as a prospective drug for the treatment and prevention of neurodegenerative diseases such as AD and Parkinson’s disease (PD). Nobiletin, as the primary active component of CRP, demonstrates various beneficial effects concerning Alzheimer’s disease (AD). Primarily, it plays a pivotal role in addressing one of AD’s key features, the deposition and aggregation of extracellular amyloid-beta (Aβ) [47]. Nobiletin reduces Aβ production, facilitates its clearance, and inhibits excessive aggregation, thereby mitigating the toxic impact of Aβ on neurons [90]. This action aids in shielding neurons from Aβ-related damage. Additionally, nobiletin in CRP influences neurotransmitter balance, particularly acetylcholine activity [51]. By enhancing acetylcholine functionality and modulating glutamate receptors, it improves neurological function and helps alleviate memory and cognitive impairments commonly observed in AD patients [11]. Moreover, nobiletin in CRP demonstrates significant antioxidative and anti-inflammatory properties. It regulates oxidative stress, reducing levels of detrimental oxidizing agents and safeguarding neurons against oxidative damage [91]. Simultaneously, it suppresses inflammatory responses, diminishing the release of inflammatory mediators, thereby protecting the nervous system from inflammatory harm [52]. Furthermore, it modulates the phosphorylation status of Tau proteins, preventing their excessive phosphorylation, thereby maintaining microtubule stability and reducing neuronal degeneration [92]. This action is crucial in preventing Tau protein-associated neural damage during the pathological progression of AD [93]. Finally, nobiletin regulates circadian rhythms, ameliorating sleep disturbances. This holds significant importance for the prevalent sleep issues seen in AD patients, aiding in enhancing their quality of life and neurological function. However, there is limited clinical research on nobiletin treatment in cases of AD, necessitating further investigation to lay the groundwork for the development of potential new drugs for AD treatment. In summary, nobiletin exerts beneficial effects on various pathological processes of AD through multiple pathways, demonstrating potential therapeutic efficacy.

### 2.6. The Protective Effect on the Skeleton

CRP and its constituents have been shown to have protective effects on the skeleton. Research has indicated that flavonoids present in CRP can enhance the generation of osteoblasts by activating the BMP/p38/Smad/Runx2 pathway, thereby safeguarding bone cells in a rat model of osteoporosis induced by ovariectomy (OVX) [55]. Toshihide Suzukid et al. observed that CRP extract can lower levels of inflammatory cytokines, monocyte chemoattractant protein-1 (MCP-1), interleukin-1β (IL-1β), and plasma creatine kinase, which are elevated due to eccentric exercise-induced skeletal muscle injury, thereby exerting a protective effect on the skeleton [94]. Dong Wook Lim et al. discovered that CRP ethanol extract significantly inhibits the loss of bone mineral density (BMD); decreases serum levels of alkaline phosphatase (ALP), type I collagen C-terminal peptide (CTx), and osteocalcin (OC); and improves lipid metabolism and bone metabolism in ovariectomized (OVX) animal models, suggesting its use as a novel osteoporosis inhibitor [95]. Guangning et al. found that the active component of CRP, nobiletin, over-activates the AMPK-PGC1-α pathway, leading to enhanced mitochondrial biogenesis, exhibiting potential for protecting muscles and bones, thereby improving exercise performance [54].

### 2.7. Anti-Allergic Properties

Food allergies are recognized as type I hypersensitivities, characterized by a clinical syndrome resulting from the release of allergic mediators by mast cells and basophils [96]. They have garnered increasing attention and focus among researchers. Considerable efforts have been made to treat diseases caused by food allergies, leading to the development of various medications [97]. However, some of these drugs come with numerous side effects, such as growth retardation, cataracts, and osteoporosis [98]. CRP is a product derived from citrus peels. This study involved the extraction of flavonoid compounds from CRP using a solvent-based approach, followed by their enrichment with polyamide [56]. The in vivo and in vitro anti-allergic activities of flavonoids were investigated. Under optimal conditions, the compounds with the highest content in the extract were hesperidin and naringin, with contents of 232.0325 and 98.7946 mg/g, respectively [56]. In vitro, RBL-2H3 cell lines were used to study the anti-allergic activity, revealing that flavonoids reduced the antigen-induced degranulation of β-hexosaminidase [56]. In vivo, the activity of flavonoids was tested using an allergic asthma model induced by ovalbumin. After treatment with different concentrations of flavonoid compounds, there was a significant reduction in the levels of IgE, IL-4, and IL-13. These findings suggest that the flavonoid compounds extracted from CRP hold potential for application in the field of anti-allergic therapy [56]. In conclusion, the flavonoid components extracted from CRP may play a crucial role in modulating B cells by reducing IgE synthesis or in influencing T cells by lowering levels of TH2-like cytokines responsible for IgE synthesis.

## 3. Application in Food

### 3.1. CRP Products

#### 3.1.1. Beverages

With the rapid evolution of food consumption patterns, consumers are increasingly seeking higher-quality, sensory-appealing foods [99]. In recent years, the excessive consumption of sugar and salt has led to obesity, diabetes, and cardiovascular diseases, making the modulation of sweetness and/or saltiness perception through appropriate aromas a global trend. Odors described as fruity, sweet, or citrusy are associated with sweetness and have the potential to enhance it [100]. Additionally, herbs are commonly regarded as enhancers of saltiness [101]. CRP, the dried and aged peel of citrus fruits or their varieties, stored for over three years, is a foodstuff with medicinal properties, making it a promising candidate [25]. As CRP ages, its freshness diminishes, but it becomes richer, making it more attractive and flavorful. CRP pairs excellently with meats and fish, imparting a fragrant aroma while reducing their gaminess with a unique bitter taste [102]. CRP is not only used in savory dishes but also preferred in sweet foods due to its evolving aroma during aging, such as “Chenpi Green Bean Soup”. Furthermore, research has indicated that tangerine peel is a rich source of vitamin C; hence, it is recommended for enriching food [102]. Sir Elkhatim et al. reported that the vitamin C content of tangerine peel is 110.4 milligrams per 100 g [103]. Researchers have found that the vitamin C content of tangerine peel is equivalent to that of the whole fruit. Teixeira et al. demonstrated that by adding orange peel to orange jam, the vitamin C content increased significantly from 124.83 milligrams per 100 g to 150.12 milligrams per 100 g [104]. The recommended daily intake of vitamin C for adults is 75–90 mg, with a tolerable upper intake level of 2 g/day for this vitamin [105]. Therefore, the vitamin C content in fortified beverages containing tangerine peel ensures an adequate daily intake of vitamin C.

#### 3.1.2. Jelly

Additionally, CRP is rich in pectin [106]. Pectin is a heteropolysaccharide naturally present in all plant tissues. It exists in varying amounts in fruit cell components (in the cell wall, serving as one of the primary agents binding cellulose microfibrils and can covalently link with other polymers). Intracellular pectin provides channels for the passage of nutrients and water [106]. Pectin (a plant coagulant) is primarily used as a gelling agent, thickener, and stabilizer in food, with its classic application being to give jams, jellies, or marmalades a jelly-like consistency; without it, they would remain sweet fruit juice [107]. As mentioned above, CRP contains a variety of bioactive components, such as volatile oils, flavonoids, alkaloids, and polysaccharides [85]. Being a natural product, CRP can be combined with other foods to create functional foods. Researchers developed a functional kiwifruit jelly containing CRP that is rich in phenolic compounds, low in calories, and has antioxidant and anti-inflammatory properties, preventing fat accumulation [108]. This functional leisure food meets modern nutritional demands well and promotes the processing and utilization of natural products, holding promising prospects in the functional food industry [108].

#### 3.1.3. Tea

Tangerine peel, also known as “Jupi” in China, is a well-known dual-use food-medicine ingredient in the production of popular Fast Moving Consumer Goods (FMCG) products [109]. Composite CRP Tea (CCT) is a popular herbal beverage made from CRP, lingzhi, and Pu’er tea in a ratio of 1:1:1 [110]. Polymethoxyflavones are the primary lipid-lowering components in CRP, improving intracellular lipid accumulation, inhibiting fat production, and regulating lipid abnormalities, with their metabolic protective effect relying on gut microbiota [111]. CCT effectively reduces diet-induced obesity, hepatic steatosis, lipid abnormalities, and insulin resistance [110]. Tangerine peel tea, an emerging beverage, is made from Pu’er tea and CRP [112]. In recent years, it has gained increasing popularity among consumers due to its potential health benefits and unique flavor. The combination of Pu’er tea and tangerine peel has attracted much attention. Pu’er tea, known for its microbial fermentation process and produced from the large leaf tea plant (Camellia sinensis (Linn.) var. assamica (Masters) Kitamura) dried in Yunnan Province, China, has been proven to have various health-promoting effects, such as its antioxidation, anti-aging, and lipid-lowering properties [113]. On the other hand, tangerine peel, especially Guangdong’s Xin Hui County “Tea Pool”-sourced CRP, is a precious ingredient widely used in cooking and traditional medicine due to its unique health benefits and flavor [6]. The production process of tangerine peel tea integrates the essence of Pu’er tea and tangerine peel, allowing the tea leaves to absorb the fruity flavor of the tangerine peel and blend with the rich taste of Pu’er tea [112]. Traditionally, both Pu’er tea and tangerine peel are believed to improve in quality with prolonged storage time [113]. For instance, only CRP stored for more than three years is considered qualified [114]. Tangerine peel tea not only continues this tradition but also innovatively combines the two, presenting a unique taste and health benefits. Although the demand for tangerine peel tea is rapidly increasing on the market, research on its chemical composition and biological activity is relatively scarce, providing more possibilities for future in-depth exploration [112].

#### 3.1.4. Essential Oil

In addition to its advanced aroma, three-year-aged CRP also contains a considerable number of essential oils [115]. Recent research has indicated that tangerine peel essential oil contains various bioactive components, effectively controlling microbial and enzymatic activity [47]. CRP is rich in essential oil active constituents, such as γ-terpinene, D-limonene, 2-thujene, 3-carene, α-pinene, β-myrcene, linalool, etc. It functions as an antitussive, expectorant, and cholelitholytic and promotes the secretion of digestive juice; it also possesses antibacterial and anti-inflammatory properties [116]. Moreover, the essential oil is utilized in toothpaste, soap, natural fragrances, food additives, and various daily products [117,118]. Additionally, CRP-extracted oil is suitable as a glazing material, enhancing the freshness of fish during superchilled storage. Research has demonstrated that oils extracted from mandarin (*Citrus reticulata*), bitter orange (*Citrus bigarradia*), and sweet orange (*Citrus sinensis*), analyzed through GC-MS, exhibit excellent effects when used as surface coatings [119]. Samples of blunt snout bream (*Megalobrama amblycephala*) stored under superchilled conditions at −1 ± 0.2 °C for 25 days showed significant inhibition of structural, electrical, moisture, chemical, microbial, textural, and sensory changes during storage. These findings suggest the effective applications of CRP essential oil in the storage of aquatic products [119].

### 3.2. Dietary Supplements

CRP holds a prominent status in both traditional Chinese medicine and contemporary clinical practice, as well as in the food industry as a seasoning and dietary supplement. In China and other Eastern countries, CRP is widely utilized not only as a dietary supplement but also as one of the most popular traditional medicinal herbs for treating various ailments. It is particularly esteemed for its efficacy in addressing indigestion and inflammatory syndromes of the respiratory tract [120]. Classified as a “top-grade” medicinal herb, CRP is renowned for its ability to clear fever, relieve chest congestion, and promote gastric health through prolonged consumption. Traditional Chinese medicine classics such as the “Ben Cao Bei Yao” and “Tang Ye Ben Cao” document CRP’s diverse therapeutic uses, while Li Shizhen’s “Ben Cao Gang Mu” notes its compatibility with tonics, purgatives, and detoxifying agents [120]. In clinical practice, CRP is frequently integrated into herbal formulations to address digestive discomfort, stomach pain, abdominal bloating, cough, and other disorders of the digestive and respiratory systems [120] It is often combined with other herbs known for promoting Qi and blood circulation, such as bitter orange peel, fingered citron, cyperus, amomum fruit, white peony, and salvia, among others. For example, formulations like the “Nu Ji Wan” containing CRP, cyperus, and white peony serve to nourish Qi and blood, promote circulation, invigorate blood, and alleviate pain. Moreover, CRP is incorporated into various prescription formulations available in forms such as an oral liquid, granules, pills, and capsules [121]. Clinical studies have demonstrated the efficacy of CRP-containing formulations in the treatment of conditions like functional dyspepsia and chronic atrophic gastritis, with significant improvements observed in symptoms and pathology [6,122].

## 4. Toxicology

CRP contains excessive moisture and polysaccharides, making it susceptible to mold growth during storage [6]. Environmental factors such as temperature and humidity provide an ideal breeding ground for fungi like Aspergillus, which, in turn, produce aflatoxins (AF). Therefore, the safety of CRP is significantly impacted by AF [123]. Currently, various methods are employed to detect AFs in CRP, including TLC, HPLC with pre- or post-column derivatization, UPLC combined with immunomagnetic bead enrichment and purification, LC/MS, fluorescence spectrophotometry, and enzyme-linked immunosorbent assays (ELISAs) [123]. For long-term storage of CRP, a key measure to prevent citrus fruit fungal growth is the use of anti-fungal edible coating technology [124]. Traditional chemical fungicides play a crucial role in preserving citrus fruits, but concerns over human health risks, environmental pollution, and the spread of pathogenic fungal strains are increasing the demand for alternative solutions. Anti-fungal edible coatings represent a new, safe technology that effectively extends the storage life of citrus fruits and reduces the risk of fungal growth. It serves as a sustainable alternative with low toxicity, environmental friendliness, and durability, while maintaining the appearance and eating quality of citrus fruits [6]. Moreover, the antifungal activity, either in vitro or in vivo, of many essential oils extracted from plants or fruits such as birch, bergamot, cumin, cinnamon, citrus, clove, lemongrass, oregano, thyme, or tea tree against major citrus pathogens has been well documented [125]. These essential oils offer natural protection against fungal pathogens and can be integrated into anti-fungal edible coatings to enhance their efficacy. By leveraging the antifungal properties of these essential oils, citrus producers can potentially reduce their reliance on synthetic chemical fungicides, addressing concerns related to human health and environmental impact [125]. For vulnerable groups such as children and pregnant women, who are more sensitive to contaminants like pesticides and mold toxins, regulatory agencies typically establish maximum residue limits (MRLs) for herbal products, including CRP, to protect public health [124]. However, due to differences in immune systems and physiological characteristics compared to adults, the biological activity and potential toxic effects of coating components require additional scrutiny and research. Therefore, ongoing research and monitoring are crucial to better understand the risks of contaminants in CRP and to develop effective strategies to reduce exposure, including improving detection methods and developing safer agricultural practices.

Moreover, caution should be exercised when using CRP for patients with Yin deficiency and dry cough, internal heat excess, or conditions involving hemoptysis. Yin deficiency, a condition characterized by a lack of nourishing fluids in the body, can lead to symptoms such as dryness and heat sensations. It is recommended to avoid consuming CRP in conjunction with cold and raw foods, as the cold nature of such foods may generate dampness and oppose the properties of CRP, potentially affecting the therapeutic efficacy of the herb [6].

## 5. Conclusions and Future Perspectives

In conclusion, this paper comprehensively explores the chemical constituents of tangerine peel (CRP) and its diverse health benefits, showcasing its potential as a multifaceted traditional Chinese medicine. Firstly, we discussed the significant therapeutic effects of CRP and its constituents in cancer treatment, particularly in lung, nasopharyngeal, liver, and breast cancers. Secondly, we elucidated CRP’s impact on cardiovascular diseases, including its interventions in myocardial injury, cardiac remodeling, and pathological cardiac hypertrophy. Additionally, we outlined CRP’s protective effects on the digestive system, such as modulating gut microbiota, promoting gastrointestinal motility, and inhibiting gastric ulcers. Furthermore, we highlighted CRP’s antioxidant and anti-inflammatory properties, as well as its potential use in Alzheimer’s disease treatments. Finally, we explored CRP’s protective effect on the skeleton, demonstrating its role in promoting osteoblast generation and inhibiting bone loss. Looking ahead, despite the promising applications of CRP and its constituents across various domains, further research is warranted. Firstly, more in-depth elucidation of the mechanisms of action of CRP’s chemical constituents is needed to better understand their roles in disease treatment. Secondly, additional clinical studies are necessary to validate the safety and efficacy of CRP and its constituents in humans. Moreover, the optimization of extraction, purification, and formulation processes for CRP is required to enhance its pharmacological activity and bioavailability. Lastly, strengthening interdisciplinary collaborations with modern medicine will facilitate exploring CRP and its constituents’ applications across diverse disciplines, providing a solid scientific basis for their widespread clinical use.

## Figures and Tables

**Table 1:** The beneficial effects of CRP on disease and its underlying mechanisms.

Diseases	Components	Health Benefits	Mechanisms	Experimental Models	Dosages	References
Lung Cancer	Hesperidin	Inhibited nicotine toxicity smoking-induced lung cancer	Reduced MMP expression and enhanced antioxidant capacity	Male albino wistar rats	25 mg/kg/day for 22 weeks	[23]
		Inhibited cancer cell growth and induce apoptosis	Inhibited NSCLC cell proliferation and promotes apoptosis via the miR-132/ZEB2 pathway	Adult Sprague-Dawley (SD) male rats (weight, 328–365 g)	60 mg/kg/day	[24]
		Developed for the treatment of non-small cell lung cancer	Induced apoptosis via the mitochondrial pathway	A549 human NSCLC cell line and BEAS-2B human normal lung epithelial cell line	/	[25]
		Reduced the risk of COPD progressing to lung cancer	Regulated AKT1, IL6, VEGFA, MMP9 and TP53	Seven-week-old female ICR mice with body weight of 23 ± 2 g (*n* = 90)	(25, 50, 100 mg/kg/day)	[26]
	Naringin	Reduced mucus production and inhibited tumor progression	Inhibited the synergistic activity of MAPKs/AP-1 and IKKs/IκB/NF-κB signal-ing pathways	A549 cells (human lung adenocarcinoma cell line)	/	[27]
		Inhibited the proliferation and apoptosis of small cell lung cancer cells	Inhibited PI3K/AKT/mTOR and NF-κB pathways	Human H69AR SCLC cell line	/	[28]
	Tangeretin	Inhibited the growth of cancer cells	Induced G1 arrest or apoptosis in human non-small cell lung cancer cells	/	/	[29]
	Tangeretin derivatives	Inhibited the growth of CL1-5 lung cancer cells	Induced G2/M cell cycle arrest, autophagy, and apoptosis	CL1-5 lung cancer cells	/	[30]
Nasopharyngeal Cancer	Nobiletin	Inhibited the growth of nasopharyngeal cancer cells	Induced C666-1 cells apoptosis	C666-1 cells	/	[30]
Liver cancer	Hesperidin	Inhibited the invasiveness of HCC cells	Inhibited NF-κB and AP-1 activities	HepG2 cells, a human hepatocellular carcinoma cell line	/	[31]
	Tangeretin	Decreased proliferation and migration of HepG2 cells	Activated the JNK pathway, reduced Bcl-2 phosphorylation	HepG2 cells, a human hepatocellular carcinoma cell line	90 μg/mL/day	[30]
	Naringin	Inhibited the growth of hepatocellular carcinoma cells	Reduced cell proliferation and induced apoptosis in liver cancer	Male Wistar rat model	(40 mg/kg BW) for 16 weeks	[32]
	Hesperidin/naringin	Induced HepG2 cells apoptosis	Inhibited NF-κB and AP-1 activities through downregulating MMP-9 expression in HCC cells	HepG2	/	[33]
Breast Cancer	Nobiletin	Inhibited tumor growth	Inhibited ERK1/2 and PI3K/AKT pathways	MDA-MB-468 cell line	/	[34]
	Tangeretin	Inhibited breast cancer cell metastasis	Targeted TP53, PTGS2, MMP9 and PIK3CA and modulated the PI3K/AKT pathway	/	/	[35]
	Hesperidin	Treated BCSCs exhibited reduced proliferation	Targeted BCSCs by inhibited the Stat3/Sox2 signaling pathway	Breast cancer cell lines MCF-7 and MDA-MB-231	2.5 mg/kg 4 times	[36]
	The combination of hesperidin and chlorogenic acid	Treated DMBA-induced breast cancer with cell transplantation	Reduced DMBA-induced oxidative stress and renal DNA damage	Seven-week-old virgin female Wistar rats	50 mg/kg for four weeks	[37]
	Naringin	Induced apoptosis and caused DNA damage	Enhanced concentration-dependent cytotoxicity against human breast cancer cell line MCF-7	MCF-7 cell line	/	[38]
		Treated drug-resistant cancer cells	Inhibited activity of hesperidin on the proliferation of MCF-7-GFP-Tubulin cells	MCF-7-GFP-Tubulin Cells	/	[39]
		Inhibited the breast cancer cell metastasis	Induced the protein kinase C-α translocation to the cell membrane by chlorogenic acid	Breast cancer cell MCF-7	100–600 µM/72 h	[40]
		Improved cell migration of MDA-MB 231 cells	Inhibited the cell proliferation, with increased p21 and decreased inhibitor	MDA-MB-231, MDA-MB-468, and BT-549 cells/Severe Combined Immunodeficiency (SCID) hairless female mice	100 mg/kg body weight	[41]
Cardiovascular diseases effects	Hesperidin	Inhibited cardiomyocyte apoptosis and reduced oxidative stress damage	Upregulated PPARγ expression	Male Wistar albino rats of either sex weighing 180–200 g	100 mg/kg/day	[42]
	Nobiletin	Alleviated myocardial dysfunction and attenuated myocardial ischemia and reperfusion injury	Induced activation and overexpressed of MMP-2 and MMP-9	Male wistar rats	10 mg kg^−1^, and 25 mg kg^−1^ for four weeks	[41]
	Naringenin	Exerted anti-ischemic effects	Achieved this through the activation of mitochondrial BK channels	Male wistar rats	/	[43]
	Naringin	Attenuated myocardial strain and inflammatory responses in sepsis-induced myocardial dysfunction	Regulated PI3K/AKT/NF-κB pathway	Adult male Sprague-Dawley (SD) rats (weight: 300 ± 5 g, age: 8 w–9 w)	50 and 100 mg/kg for 7 days	[44]
	Extract of Citri reticulatae Pericarpium (CRP)	Protected the angiotensin II (Ang II)-induced pathologic cardiac hypertrophy	Involved the activation of PPARγ, with Peroxisome Proliferator-Activated Re-ceptors (PPARs)	Eight-week-old C57BL/6J male mice	0.5 g/kg/d for 4 weeks	[45]
		Improved cardiac function induced by Ang II infusion	Decreased in collagen I protein levels	Wild type mice	/	[45]
Effect on the Digestive System	Hesperidin	Showed synergism in anti-ulcer activity	Regulated intestinal flora and inhibits intestinal smooth muscle contractions	Thirty Wistar albino rats of either sex weighing 200–250 g	100 mg/kg	[46]
		Promoted gastrointestinal motility	Increased levels of acetylcholine (ACh) and motilin (MTL), and decreased levels of Substance P (SP) and vasoactive intestinal peptide (VIP)	/	/	[46]
	Ethyl acetate	Demonstrated the ability to enhance gastrointestinal motility	/	/	/	[46]
Antioxidant and Anti-inflammatory Effects	Nobiletin	Inhibited anti-inflammatory activity	Used a lipopolysaccharide (LPS)-activated BV2 microglia culture system	The BV2 microglial cells	25–100 μM	[47]
		Inhibited anti-inflammatory activity	Inhibited NO production in lipopolysaccha-ride (LPS)-activated Raw 264.7 murine macrophage cells	BV-2 microglial cells	/	[48]
	Tangeretin	Suppressed itching caused by allergies	Inhibited the action of histamine and the activation of nuclear factor-κB (NF-κB), activator protein (AP)-1	Male ICR mice (5 weeks old, 20–25 g) and male Hartley guinea pigs (270–330 g)	nobiletin 5 mg/kg, tangeretin 10 mg/kg	[49]
		Regulated inflammatory responses by modulated the activity of NF-κB via various signaling pathways	Inhibited LPS-induced phosphorylation of MAPKs and Akt in BV2 cells	BV2 cells	/	[50]
Alzheimer’s disease (AD)	Nobiletin	Prevented cerebrovascular lesions	Reduced the abnormal accumulation of neurotoxic amyloid-beta peptides	Mice	/	[11]
		Exerted neuroprotective effects	Improved cognitive impairment and pathological features in animal models of AD	APP-SL 7–5 Tg mice, olfactory bulbectomized mice and 3XTg-AD mice	/	[51]
		Suppressed neurasthenia	Achieved neuroprotection through anti-inflammatory, neurotrophic, and cholinergic effects	APP-SL 7–5 Tg mice, olfactory bulbectomized mice and 3XTg-AD mice	/	[51]
		Prevented neuroinflammation	Prevented the mRNA expression of inducible NO synthase (iNOS) and cy-clooxygenase-2 (COX-2), respectively	BV-2 cells	/	[52]
		Prevented neuroinflammation	Inhibited the LPS-induced mRNA expression of CCL2, CXCL1, IL-6, and TNFα	BV-2 cells	nobiletin (50, 100 μM)	[52]
	Tangeretin	Prevented cerebrovascular lesions	Reversed N-methyl- D-aspartate (NMDA) receptor hypofunction	Mice	/	[11]
The protective effect on the skeleton	Nobiletin	Restored bone mass and benefit bone health	Inhibited the NF-κB pathway	OVX mice	nobiletin (60 μM)	[53]
	Tangeretin	Improved exercise performance	Activated mitochondrial biogenesis signaling pathway	C2C12 myoblasts/Male Kunming mice	100 mg/kg tangeretin	[54]
		Improved exercise performance	Enhanced mitochondrial biogenesis via activating the AMPK-PGC1-α pathway	C2C12 myoblasts/Male Kunming mice	/	[55]
Other effects (Food allergy and so on)	Hesperidin and Narirutin	Decreased the levels of TH2-like cytokines responsible for IgE synthesis	Reduced releases of antigen-induced beta-hexosaminidase degranulation	RBL-2H3 cell line/Female BALB/c mice (6–8 weeks old, 20–25 g)	232.0325 and 98.7946 mg/g	[56]

## Data Availability

The original contributions presented in the study are included in the article, further inquiries can be directed to the corresponding author.
